# Evaluation of the effects of dexmedetomidine infusion on oxygenation and lung mechanics in morbidly obese patients with restrictive lung disease

**DOI:** 10.1186/s12871-018-0572-y

**Published:** 2018-08-14

**Authors:** Ahmed Hasanin, Kareem Taha, Bassant Abdelhamid, Ayman Abougabal, Mohamed Elsayad, Amira Refaie, Sarah Amin, Shaimaa Wahba, Heba Omar, Mohamed Maher Kamel, Yaser Abdelwahab, Shereen M. Amin

**Affiliations:** Department of anesthesia and critical care medicine, Cairo university, 01 elsarayah street, Elmanyal, Cairo, 11559 Egypt

**Keywords:** Dexmedetomidine, Morbidly obese, Oxygenation

## Abstract

**Background:**

Dexmedetomidine infusion improves oxygenation and lung mechanics in patients with chronic obstructive lung disease; however, its effect in patients with restrictive lung disease has not been thoroughly investigated yet. The aim of this work was to evaluate the effects of dexmedetomidine infusion on oxygenation and lung mechanics in morbidly obese patients with restrictive lung disease.

**Methods:**

Forty-two morbidly obese patients scheduled for bariatric surgery were included in the study. Patients were randomized to receive either dexmedetomidine infusion at a bolus dose of 1mcg/Kg followed by infusion at 1 mcg/Kg/hour for 90 min (Dexmedetomidine group), or normal saline infusion (Control group). Both groups were compared with regard to: oxygenation {P/F ratio: PaO_2_/fraction of inspired oxygen (FiO_2_)}, lung compliance, dead space, plateau pressure, blood pressure, and heart rate.

**Results:**

Dexmedetomidine group showed significant improvement of the PaO_2_/FiO_2_ ratio, and higher lung compliance compared to control group by the end of drug infusion. Dexmedetomidine group demonstrated decreased dead space, plateau pressure, blood pressure, and heart rate compared to control group by the end of drug infusion.

**Conclusion:**

A 90-min dexmedetomidine infusion resulted in moderate improvement in oxygenation and lung mechanics in morbidly obese patients with restrictive lung disease.

**Trial registration:**

clinicaltrials.gov: NCT02843698 on 20 July 2016.

## Background

Dexmedetomidine is a selective α − 2 agonist with various clinical uses in both anesthesia and intensive care unit. In addition to its sedative and cardiovascular effects, dexmedetomidine has favourable respiratory effects in animals [1, 2], and in selected patient groups in humans. The effects of dexmedetomidine on oxygenation and lung mechanics had been investigated in obstructive lung disease. Dexmedetomidine decreased dead space and improved both lung compliance and oxygenation in chronic obstructive pulmonary disease (COPD) patients undergoing lung cancer surgery [3].

In restrictive lung disease, the possible effect of dexmedetomidine was not well investigated in humans. In an animal model of experimental obesity, dexmedetomidine administration showed better morphological and functional lung characteristics compared to propofol [4]; thus, we hypothesized that a similar effect could be present in humans. Morbidly obese patients are characterized by the high prevalence of restrictive lung disease [5]; thus, we investigated the effects of dexmedetomidine infusion on oxygenation (P/F ratio) as well as lung mechanics (compliance and dead space) in a selected group of morbidly obese patients with restrictive lung disease, to find out the possible benefits of this drug on this population.

## Methods

A randomized controlled double-blinded study was conducted in Cairo university hospital after institutional board review approval (N − 12-2016) on 24 December 2016. The study was registered at clinical.trials.gov registry system on 20 July 2016 (NCT02843698). A written informed consent was obtained from participants before recruitment. Patients were randomized according to an online random number generator. Concealment was achieved using sealed opaque envelopes.

The study included 42 morbidly obese patients {with body mass index (BMI) above 40 Kg/m^2^} with restrictive lung disease {diagnosed by pulmonary function tests: forced vital capacity (FVC) < 70%}, scheduled for laparoscopic sleeve gastrectomy. Exclusion criteria were: heart failure, arrhythmias, severe liver or kidney impairment. Patients with forced expiratory volume in 1 sec (FEV1)/FVC < 70% were also excluded.

Upon arrival at the operating room, patients were pre-medicated with metoclopramide (10 mg) and ranitidine (50 mg). Basic monitors were applied {electrocardiogram (ECG), non-invasive blood pressure monitor, capnography and pulse oximetry}. Drug dosing was calculated using lean body weight for all drugs except neostigmine (Total body weight was used) [6]. LBW was calculated using James equation {Men: (1.10 weight) - (128 (weight/height)^2^}, {Women: (1.07 weight) - (148 (weight/height)^2^} [6]. Anesthesia was induced by propofol (2 mg/Kg LBW) and fentanyl (2 μg/Kg LBW). After induction of anesthesia, endotracheal tube was inserted aided by rocuronium (0.5 mg/Kg LBW). Anesthesia was maintained by isoflurane (1–1.5%) and rocuronium (0.1 mg/Kg/40 min) at FiO_2_ of 40%. Intravenous paracetamol (2 g) and Ketorolac (40 mg) was administered before the end of surgery.

Patients were mechanically ventilated using Maquet (Flow-i) anesthesia machine. Our ventilatory management included: volume controlled ventilation, low tidal volume (6-8 ml/Kg LBW), positive end expiratory pressure (PEEP) 8–10 mmH_2_O, and respiratory rate adopted to maintain end-tidal CO_2_ between 30 and 35 mmHg [7]. No recruitment manoeuvres were used in our patients.

Patients were randomly allocated into our double-blinded study using computer generated sequence into two groups: 1- Dexmedetomidine group (*n* = 21): received dexmedetomidine (Precedex, Hospira, Lake forest, IL, USA) in a dose of (1μg/Kg LBW) bolus 15-min after endotracheal intubation, followed by 0.5μg/Kg/hour continuous infusion for 90 min. 2- Control group (*n* = 21): received 1 mL normal saline followed by continuous normal saline infusion for 90 min. The study drug was prepared and the infusion rate was calculated by a research assistant to ensure blinding of the investigator.

At the end of the operation, isoflurane was discontinued, residual neuromuscular block was reversed using neostigmine (0.05 mg/Kg) and atropine (0.02 mg/Kg), then patient was extubated. In the post anesthesia care unit (PACU), patient was monitored by basic monitors (pulse oximetry, non-invasive blood pressure monitor, and ECG). Patients with oxygen saturation less than 88% on 6-l oxygen mask were admitted to the intensive care unit.

Dynamic lung compliance was calculated using Maquet anesthesia machine as: tidal volume/ (peak airway pressure - PEEP). Static lung compliance was calculated as: Tidal volume/ (plateau pressure - PEEP). Plateau pressure was calculated by increasing the end-inspiratory pause to 30–40%. Physiological dead space was calculated by Hardman & Aitkenhead equation [8]: Vd/Vt = 1.14 (PaCO_2_–EtCO_2_)/PaCO_2_–0.005.

### Primary outcome

Oxygenation by the end study drug infusion (assessed by P/F ratio: PaO_2_/Fraction of inspired oxygen).

### Secondary outcomes

- Intraoperative oxygenation, lung compliance (static and dynamic), dead space (Vd/VT), and PaCO_2_ (5 minutes after endotracheal intubation and positioning, at 45 min, and at 90 min after starting drug infusion).
- Heart rate, Systolic blood pressure (SBP), diastolic blood pressure (DBP), and plateau airway pressure (At the baseline, then every 15 min for 2 h).
- Demographic data (age – gender - BMI – smoking – comorbidities), operative data (surgical time – intraoperative fluids – blood loss), the need for postoperative ICU, the need for postoperative mechanical ventilation, postoperative complications, and Pasero opioid-induced sedation scale [9].
- The change in each of P/F ratio and dynamic lung compliance (Δ P/F ratio: P/F ratio by the end of the study drug infusion – P/F ratio at the baseline, Δ compliance: dynamic lung compliance by the end of the study drug infusion – dynamic lung compliance at the baseline)
- Δ dead space (%): dead space by the end of the study drug infusion – dead space at the baseline/dead space by the end of the study drug infusion %).

### Sample size calculation

In a pilot study on 10 patients, we reported an intraoperative P/F ratio of 310 ± 32 in morbidly obese patients undergoing bariatric operations. Using MedCalc Software version 14.10.2 (MedCalc Sofware bvba, Ostend, Belgium), we calculated a conservative sample size that could detect 10% difference in P/F ratio (i.e. 31) between the two study groups. A minimum number of 36 patients (18 patients per group) was calculated to have a study power of 80% and alpha error of 0.05. The number was increased to 41 patients (21 patients per group) to compensate for possible drop-outs.

### Statistical analysis

Statistical package for social science (SPSS) software, version 15 for Microsoft Windows (SPSS inc., Chicago, iL, USA) was used for data analysis. Categorical data (incidences of complications and comorbidities) were presented as frequency (%) and analysed by chi square test. Continuous data were checked for normality using Shapiro-Wilk test and was presented as mean (standard deviation) or median (interquartile range) as appropriate. Continuous data (PF ratio, compliance, dead space, age, blood loss, etc) were analysed using unpaired t test or Mann Whitney as appropriate. Repeated measures (blood pressure, heart rate, plateau pressure) were analysed using analysis of variance (ANOVA) for repeated measures with post-hoc pairwise comparisons using the Boneferroni test. A *P* value less than 0.05 was considered statistically significant.

## Results

**Fig. 1:**
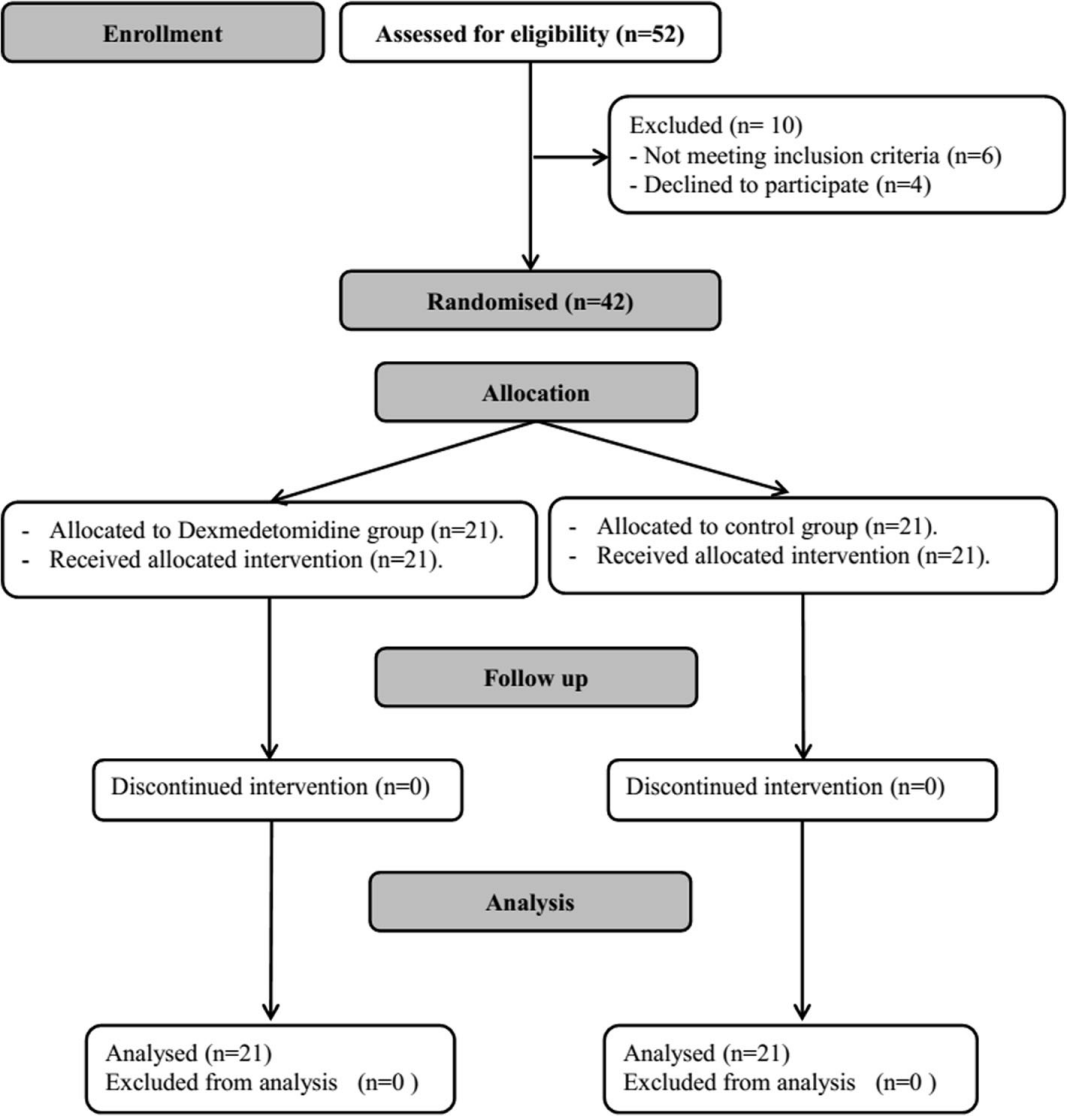
Patient recruitment chart

Forty-two patients were available for final analysis (Fig. 1). Demographic data and baseline characteristics were comparable between both study groups (Table 1). Pre-operative pulmonary functions were also comparable between the two study groups (Table 1). At 90 min, there was significant improvement in both P/F ratio and lung compliance within dexmedetomidine group compared to the baseline reading. Δ P/F ratio was significantly higher in dexmedetomidine group compared to control group {32 (43) mmHg versus − 2 (60) mmHg, *P* = 0.046) (Table 2).

**Table 1:** Demographic data, baseline characteristics, and patient outcomes. Data are presented as mean (standard deviation), median (quartiles), and frequency (%)

	Control group (*n* = 21)	Dexmedetomidine group (*n* = 21)	*P* value
Age (years)	32 (5)^a^	29 (4)	0.05
BMI (kg/m^2^)	45 (3)^a^	48 (4)	0.03
Smoking	5 (25%)	6 (29%)	0.95
COPD, emphysema	0(%)	0 (0%)	1
FVC (% predicted)	65 (61,67)	62 (60,66)	0.28
FEV1/FVC (%)	81 (78,84)	80 (77,83)	0.33
Baseline PaCO_2_ (mmHg)	42 (7)	39 (5)	0.1
Surgical duration	113 (19)	109 (18)	0.5
Intraoperative fluids	844 (101)	831 (103)	0.7
Blood loss	238 (76)	258 (78)	0.4
Postoperative complications			
- ICU admission	3 (14%)	2 (10%)	0.9
- Invasive ventilation	2 (10%)	1 (5%)	1
- Anastomotic leakage	0 (0%)	0 (0%)	1
- Bleeding	0 (0%)	0 (0%)	1
- Pneumonia	0 (0%)	0 (0%)	1
- 28-day death	0 (0%)	0 (0%)	1
POSS	2 (1,2)	2 (1,2)	1

*BMI* Body mass index, *COPD* Chronic obstructive pulmonary disease, *FEV1* Forced expiratory volume in the first second, *FVC* Forced vital capacity, *ICU* intensive care unit, *POSS* Pasero opioid-induced sedation scale, *P/F ratio* PaO2/fraction of inspired oxygen

^a^denotes statistical significance between both groups

**Table 2:** Lung mechanics and gas-exchange data. Data are presented as mean (standard deviation) and median (quartiles)

	Control group (*n* = 21)	Dexmedetomidine group (*n* = 21)	*P* value
P/F ratio			
- Baseline	- 305 (68)	- 298 (52)	- 0.7
- 45 min	- 300 (45)	- 305 (54)	- 0.7
- 90 min	- 303 (59)	- 330 (61)^b^	- 0.2
- Delta P/F ratio	- -2 (60)^a^	- 32 (43)	- 0.046
Dynamic compliance (mL cmH_2_O^− 1^)			
- Baseline	- 35.6 (7)	- 40 (9)	- 0.07
- 45 min	- 34.6 (5)^a^	- 41.3 (8)	- 0.004
- 90 min	- 34.5 (6)^a^	- 44.5 (9)^b^	- < 0.001
- Delta compliance	- -1 (4)^a^	- 4 (4)	- < 0.001
Static compliance (mL cmH_2_O^− 1^)			
- Baseline	- 45 (12)	- 51 (14)	- 0.14
- 45 min	- 44 (9)	- 51 (12)	- 0.057
- 90 min	- 45 (11) ^a^	- 54 (13)	- 0.01
- Delta compliance	- −0.7 (7)	- 3 (8)	- 0.1
Dead space (%)			
- Baseline	- 18 (6)	- 20 (8)	- 0.3
- 45 min	- 21 (8)^b^	- 19 (6)	- 0.4
- 90 min	- 22 (8)^a, b^	- 17 (5)^b^	- 0.02
- Delta dead-space %	- 3.4 (0.3,6.5)^a^	- − 1.6(− 8,1.5)	- 0.01
PaCO_2_ (mmHg)			
- Baseline	- 42.3 (7)	- 39.5 (5)	- 0.1
- 45 min	- 45.7 (7)^b^	- 42 (7)^b^	- 0.08
- 90 min	- 46.8 (6)^b^	- 43 (7)^b^	- 0.052
- Delta PaCO_2_	- −4.4 (6)	- −3.5 (6)	- 0.6

P/F ratio: PaO2/fraction of inspired oxygen. Delta: 90-min measurement – baseline measurement

^a^denotes statistical significance between both groups

^b^denotes statistical significance compared to the baseline reading within the same group

Compared to control group, dexmedetomidine group showed higher dynamic lung compliance at both 45 min {41.3 (8) mL cmH_2_O^− 1^ Vs 34.6 (5) mL cmH_2_O^− 1^, *P* = 0.004}, and 90 min {44.5 (9) mL cmH_2_O^− 1^ Vs 34.5 (6) mL cmH_2_O^− 1^, *P* < 0.001}, in addition to higher Δ dynamic lung compliance {4 (4) mL cmH_2_O^− 1^ versus − 1 (4) mL cmH_2_O^− 1^, *P* = 0 < 0.001} (Table 2). Static lung compliance was higher in dexmedetomidine group compared to control group at 90 min {54 (13) mL cmH_2_O^− 1^ Vs 45 (11) mL cmH_2_O^− 1^, *P* = 0.01}; whilst, both groups showed comparable static lung compliance at other time points (Table 2).

**Fig. 2:**
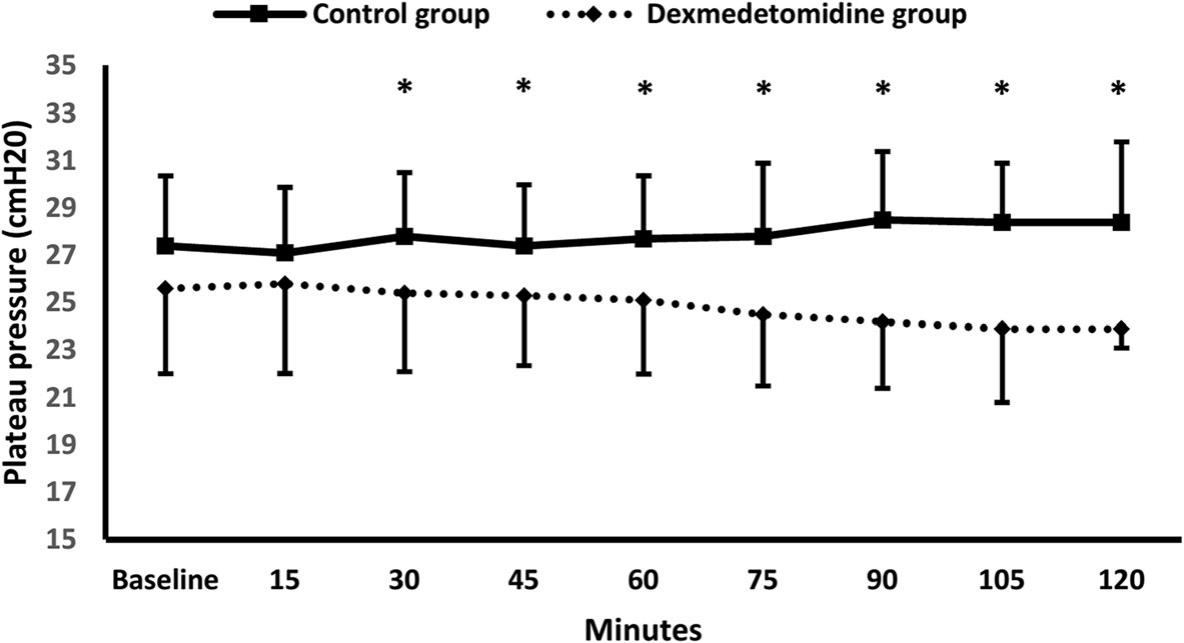
Plateau pressure. * denotes significance between both groups

Dead space significantly increased in the control group at 45 min and 90 min compared to the baseline; whilst, dead space decreased in dexmedetomidine group at 90 min compared to the baseline measurement and to the control group (Table 2). Δ dead space (%) was significantly lower in Dexmedetomidine group compared to control group {− 1.6(− 8, 1.5) % Vs 3.4(0.3, 6.5) %, *P* = 0.01} (Table 2). Plateau pressure was lower in dexmedetomidine group compared to the control group at most measurements (Fig. 2).

**Fig. 3:**
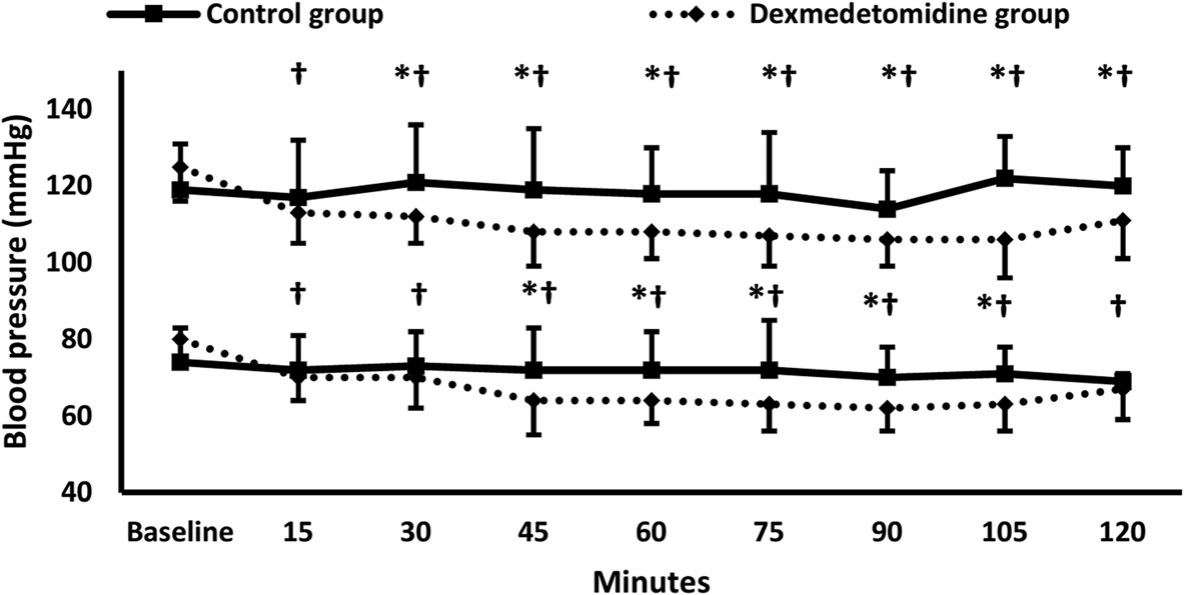
Systolic and diastolic blood pressure. * denotes significance between both groups, † denotes significance compared to the baseline reading within dexmedetomidine group

**Fig. 4:**
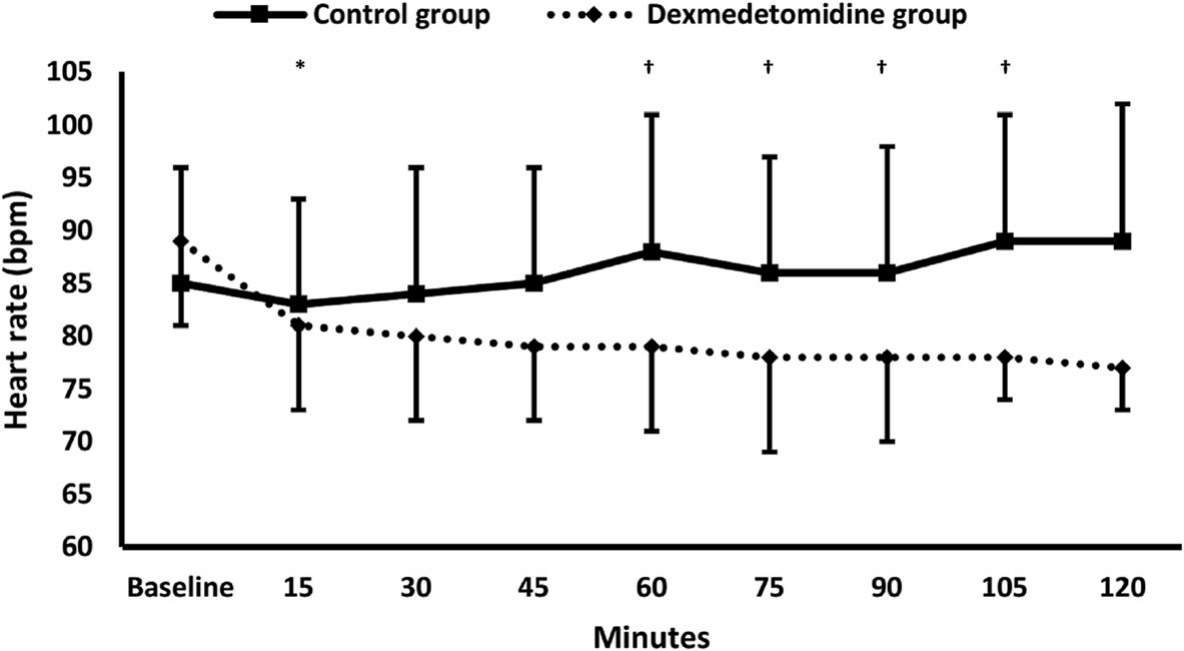
Heart rate. * denotes significance between both groups, † denotes significance compared to the baseline reading within dexmedetomidine group

Both SBP and DBP were lower in dexmedetomidine group compared to control group and compared to the baseline in most measurements (Fig. 3). Heart rate was also lower in Dexmedetomidine group compared to control group at 15 min, and to the baseline at 60, 75, 90, and 105 min (Fig. 4).

## Discussion

Dexmedetomidine infusion improved oxygenation and lung mechanics in morbidly obese patients with restrictive lung disease. Dexmedetomidine group showed higher P/F ratio, higher compliance, lower dead space, and lower plateau pressure compared to control group.

The evidence on the possible mechanism of the favourable respiratory effects of dexmedetomidine is not clear; however various mechanisms might contribute in these effects. Animal studies reported that dexmedetomidine has a bronchodilator effect in histamine-mediated bronchospasm [1]. Dexmedetomidine was reported to preserve the hypoxic pulmonary vasoconstriction from the inhibitory effect of inhalational anesthetic agents [10], increase the perfusion of ventilated lungs [10], reduce the oxidative stress [11], and increase nitric oxide (NO) [11, 12] during OLV. Dexmedetomidine enhances hypoxic pulmonary vasoconstriction through stimulation of alpha-2B receptors in vascular smooth muscles; thus, it would improve ventilation/perfusion ratio and consequently improve oxygenation [12]. As it increases the NO level in blood, dexmedetomidine reduces intrapulmonary shunt [12]. Down regulation of various inflammatory mediators might contribute to the protective respiratory effects of dexmedetomidine [13, 14]; however, this assumption needs further evaluation in more studies because we did not measure these mediators in our patients. The potential better sedation state with dexmedetomidine administration might also contribute for improvement of lung mechanics due to better relaxation of the chest wall.

When used as a sedative agent, dexmedetomidine resulted in better patient-ventilator synchrony [15] and more ventilator free hours [16] in critically ill adults. Dexmedetomidine had shortened the weaning process in critically ill children [17]. Dexmedetomidine improved oxygenation and lung mechanics during OLV in thoracic surgery [10, 12] and in COPD patients undergoing lung cancer surgery [3]. Dexmedetomidine had a protective effect against independent lung injury during OLV [13]. Dexmedetomidine improved alveolar oxygenation when used for induction of anesthesia in children with tetralogy of Fallot [18]. In restrictive lung diseases, the available data were extracted from animal studies. Compared to Propofol, dexmedetomidine administration resulted in better lung characteristics in rats with experimental obesity [4]. Dexmedetomidine had a protective effect lipopolysaccharide-induced lung injury rats [14, 19]. To the best of our knowledge, this study is the first to investigate the effect of dexmedetomidine on oxygenation and lung mechanics in patients with restrictive lung disease.

According to the baseline characteristics, all our patients showed isolated pattern of restrictive lung disease without the evidence of obstructive pattern. Thus, our findings declare a relatively novel effect of dexmedetomidine infusion on respiratory mechanics in the population of morbidly obese patients with restrictive lung disease. This effect was not previously reported in humans. The favourable effects for dexmedetomidine on oxygenation and lung mechanics in our patients were moderate; we reported an improvement of nearly 10% in the P/F ratio after 90-min dexmedetomidine infusion. Lee et al. had reported similar improvement in oxygenation and lung mechanics in COPD patients undergoing lung cancer surgery [3]. Longer duration (and/or higher doses) might result in more significant results. Moreover, this moderate improvement might be of higher value in patients with compromised respiratory status in operating room and intensive care unit. However, this assumption need to be confirmed by randomized controlled trials.

Only five of our patients (12%) experienced postoperative hypoxia in the PACU that needed ICU admission. Although there was no significant difference between both study groups regarding postoperative ICU need, we could not generalize this finding because our study was not powered enough to judge it.

### Study limitations

Our study had some limitations. First: we used only one dose of dexmedetomidine for 90 min. This was because we considered our study as an exploratory study for the effect of dexmedetomidine in this population. Based on our results, we recommend more studies to investigate whether these effects could differ with different doses and with longer infusion duration. Second: the use of Hardman & Aitkenhead equation for calculation of physiological dead space; this was because volumetric capnography was not available in our unit. Third: We did not use lung recruitment manoeuvres; this was based what Defresne et al. had reported in morbidly obese patients during laparoscopic gastric bypass surgery [20].

## Conclusion

A 90-min dexmedetomidine infusion resulted in moderate improvement in oxygenation and lung mechanics in morbidly obese patients with restrictive lung disease.

## Abbreviations

ANOVA: Analysis of variance
BMI: Body mass index
COPD: Chronic obstructive pulmonary disease
DBP: Diastolic blood pressure
ECG: Electrocardiography
EtCO2: End-tidal carbon dioxide
FEV1: Forced expiratory volume in the first second
FVC: Forced vital capacity
NO: Nitric oxide
OLV: One lung ventilation
P/F ratio: PaO_2_/Fraction of inspired oxygen
PaCO2: Pressure of arterial carbon dioxide
PACU: Post anaesthesia care unit
PaO_2_: Pressure of arterial oxygen
PEEP: Positive end expiratory pressure
SBP: Systolic blood pressure
SPSS: Statistical package for social science
